# Evaluation of the introduction of a single-lead ECG device and digital cardiologist consultation platform among general practitioners in the Netherlands

**DOI:** 10.1017/S1463423624000057

**Published:** 2024-04-18

**Authors:** Evert P.M. Karregat, Marlou A. de Koning, Jelle C.L. Himmelreich, David W. Koetsier, Jonas S.S.G. de Jong, Eric P. Moll van Charante, Ralf E. Harskamp, Wim A.M. Lucassen

**Affiliations:** 1 Department of General Practice, Amsterdam Public Health, Amsterdam University Medical Centers location AMC, University of Amsterdam, Amsterdam, the Netherlands; 2 Regionale Organisatie Huisartsen Amsterdam, Amsterdam, the Netherlands; 3 Department of Cardiology, Onze Lieve Vrouwe Gasthuis, Amsterdam, the Netherlands; 4 Department of Public & Occupational Health, Amsterdam UMC, Amsterdam Public Health, Research Institute, University of Amsterdam, Amsterdam, the Netherlands

**Keywords:** digital consultation platform, electrocardiography, implementation, primary care, single-lead

## Abstract

**Aim:**

To evaluate the use of a single-lead electrocardiography (1L-ECG) device and digital cardiologist consultation platform in diagnosing arrhythmias among general practitioners (GPs).

**Background:**

Handheld 1L-ECG offers a user-friendly alternative to conventional 12-lead ECG in primary care. While GPs can safely rule out arrhythmias on 1L-ECG recordings, expert consultation is required to confirm suspected arrhythmias. Little is known about GPs’ experiences with both a 1L-ECG device and digital consultation platform for daily practice.

**Methods:**

We used two distinct methods in this study. First, in an observational study, we collected and described all cases shared by GPs within a digital cardiologist consultation platform initiated by a local GP cooperative. This GP cooperative distributed KardiaMobile 1L-ECG devices among all affiliated GPs (*n* = 203) and invited them to this consultation platform. In the second part, we used an online questionnaire to evaluate the experiences of these GPs using the KardiaMobile and consultation platform.

**Findings:**

In total, 98 (48%) GPs participated in this project, of whom 48 (49%) shared 156 cases. The expert panel was able to provide a definitive rhythm interpretation in 130 (83.3%) shared cases and answered in a median of 4 min (IQR: 2–18). GPs responding to the questionnaire (*n* = 43; 44%) thought the KardiaMobile was of added value for rhythm diagnostics in primary care (*n* = 42; 98%) and easy to use (*n* = 41; 95%). Most GPs (*n* = 36; 84%) valued the feedback from the cardiologists in the consultation platform. GPs experienced this project to have a positive impact on both the quality of care and diagnostic efficiency for patients with (suspected) cardiac arrhythmias. Although we lack a comprehensive picture of experienced impediments by GPs, solving technical issues was mentioned to be helpful for further implementation. More research is needed to explore reasons of GPs not motivated using these tools and to assess real-life clinical impact.

## Introduction

When a general practitioner (GP) suspects a cardiac arrhythmia and finds an abnormal heart rhythm on auscultation or pulse palpation, a direct electrocardiographic registration with a resting 12-lead electrocardiogram (ECG) is warranted. Previous research showed cardiac arrhythmias were detected in nearly one-third of cases suspected of an arrhythmia in primary care, resulting in an incidence of 2.6 arrhythmias per 1000 listed patients per year (Zwietering *et al*., 1996). Unfortunately, a 12-lead (12L-)ECG is not always at direct disposal, for instance during house calls. An important arrhythmia that can be missed when obtaining an ECG at a later time is atrial fibrillation (AF), which can occur in a paroxysmal form (Raviele *et al*., 2011; Hindricks *et al*., 2020). Timely treatment of AF will lower the risk of associated complications, such as stroke and heart failure(Almutairi *et al*., 2017). Since the incidence of AF is rising, with currently already lifetime risks of about one in three among whites and about one in five among African Americans, it is important to have convenient and reliable diagnostics disposable (Kornej *et al*., 2020; Mou *et al*., 2018).

A solution to lower this diagnostic threshold could be the use of a handheld single-lead (1L-)ECG device. Such a device is able to produce a 1L-ECG signal, corresponding to lead I on a 12L-ECG recording. When equipped with a display or with an instant smartphone connection, the 1L-ECG recording can be directly visualized, and one is able to immediately assess the recording. This also offers the option to easily share the recording with colleagues or experts. Such 1L-ECG devices showed to be highly accurate in diagnosing AF when interpreted by expert readers (Duarte *et al*., 2019; Himmelreich *et al*., 2019). While GPs safely ruled out AF (negative predictive value: 98.8%), they misclassified AF in about half of suspected cases (positive predictive value: 45.7%) (Karregat *et al*., 2021). Therefore, 1L-ECG devices offer a promising tool for accessible and immediate ECG recordings, as long as GPs have access to an expert reader for consultation. To our knowledge, few scientific reports have been published on real-life implementation of 1L-ECG devices in primary care and GPs’ experiences using both these devices and a consultation platform that lowers the threshold for cardiologist interpretation.

In this study, we evaluated the real-life use of a smartphone-connected single-lead ECG device (KardiaMobile) and digital cardiologist consultation platform in diagnosing arrhythmias among GPs in Amsterdam. A digital consultation platform is only valuable whenever the consulted experts feel confident they are able to give a certain interpretation of the 1L-ECG device. If the proportion of interpretable 1L-ECGs is low, the added value of such a platform is also limited. Furthermore, such a digital consultation platform should not interfere too much with a GP’s normal consultation program. Therefore, in order for this consultation program to be an appropriate solution, the response of the consulted experts should not take too much time.

Our first aim was to investigate how often the expert panel reports being able to interpret the 1L-ECG recordings shared by GPs in the consultation platform and how fast they replied to a submitted ECG. Our second aim was to explore the experiences of this group of early-adopting GPs in using both the 1L-ECG device and digital consultation platform.

## Methods

### Study design

This work used two distinct methods to evaluate the introduction of a 1L-ECG device and digital cardiologist consultation platform among GPs. In the first – observational – part of the study, we described the interpretation of 1L-ECGs by the expert panel in the digital consultation platform. In the second part of the study, we present the results of an online questionnaire distributed among GPs to evaluate their experiences using the KardiaMobile 1L-ECG device and the digital consultation platform.

We reported the observational part of this study in accordance with the Strengthening the Reporting of Observational Studies in Epidemiology (STROBE) statement (see Supplemental Methods for checklist) (von Elm *et al*., 2008).

### Organization of (primary) health care in the Netherlands

All residents of the Netherlands are registered at one GP practice. Primary care is fully covered by the comprehensive healthcare insurance packages, mandatory for all residents, which are provided by mainly not-for-profit private, competitive health insurers (Zorginstituut Nederland, 2023). Whenever someone has a health-related question, the first person to visit is the GP. A referral from a GP is required before specialist consultation; otherwise, health insurance will not cover the costs – with exceptions to acute care.

When patients visit their GP with symptoms indicative of a possible cardiac arrhythmia, a 12L-ECG can be immediately recorded when the GP is in the possession of a 12L-ECG machine. Recent work indicated that five out of six GPs have their own in-house 12L-ECG device, and almost all GPs are able to order a 12L-ECG without cardiologist interference (Verbiest-van Gurp *et al*., 2019). GPs who record or order a 12L-ECG are responsible for the interpretation of these 12L-ECGs. Whenever the GP is unsure about the ECG interpretation, there is no common infrastructure to easily consult a cardiologist. GPs are able to refer a patient to secondary care for a 12-lead ECG with a cardiologist’s interpretation or could call the on-call cardiologist to discuss their question on an ECG’s interpretation. However, this can be experienced as disruptive to the daily workflow, and no standardized reimbursement plan is available for telephonic cardiologist consultations. Regional initiatives, such as the one described in the current work, are therefore real-world answers to the perceived need for more easily available and more structured cardiologist consultation for routine primary care ECGs. The structural use of 1L-ECG as seen in the currently described project was not common in the Netherlands at the time of the initiative itself and was an additional effort by the initiating GP cooperative to further lower the threshold for rhythm recordings.

### 1L-ECG implementation project and participating GPs

In December 2019, a GP cooperative in Amsterdam, the Netherlands (the ROHA: Regional Organization of GPs in Amsterdam), initiated a quality improvement project to raise awareness for timely AF detection among affiliated GPs. As part of this project, all 203 affiliated GPs – all having their own GP practice – who are responsible for the day-to-day care of 360,000 patients, received a free KardiaMobile 1L-ECG device sponsored by the GP cooperative between December 2019 and August 2020. To offer GPs the possibility to easily consult an expert reader, the GP cooperative simultaneously initiated a group chat within Siilo Messenger (Siilo, Amsterdam, the Netherlands), an online secured instant messaging platform designed for and restricted to healthcare professionals. The Siilo app can be accessed by smartphone and works similar to other instant messaging services, for example, WhatsApp. During the study year, the GP cooperative promoted the adhesion of this project on several occasions, with notifications in newsletters and during personal practice visits.

### Expert panel

An expert panel consisting of two cardiologists and a specialized cardiac nurse practitioner was available for consultation. This expert panel was recruited by the GP cooperative from their professional network. One cardiologist works in a public hospital in the center of Amsterdam (Onze Lieve Vrouwe Gasthuis), the other in a private clinic (Cardiologie Centra Nederland, Netherlands), and the specialized nurse in another private clinic (Stichting Cardiologie Amsterdam). They received the 1L-ECGs in the Siilo app and assessed the 1L-ECG as soon as possible. The expert panel answered on a ‘routine care’ basis; no protocols were made for a structured assessment of the shared cases. Whether the submitted 1L-ECGs were of sufficient quality to be interpreted by the expert panel, we investigated the proportion of 1L-ECGs the expert panel felt being able to interpret. For a successful implementation of the consultation platform, a quick response to the submitted ECG is important because GPs might submit the ECG during consultation. Therefore, we registered the time in which the GPs received an answer from the cardiologist. The expert panel received no reimbursement for their assessments.

### The KardiaMobile device and smartphone app

**Figure 1.**
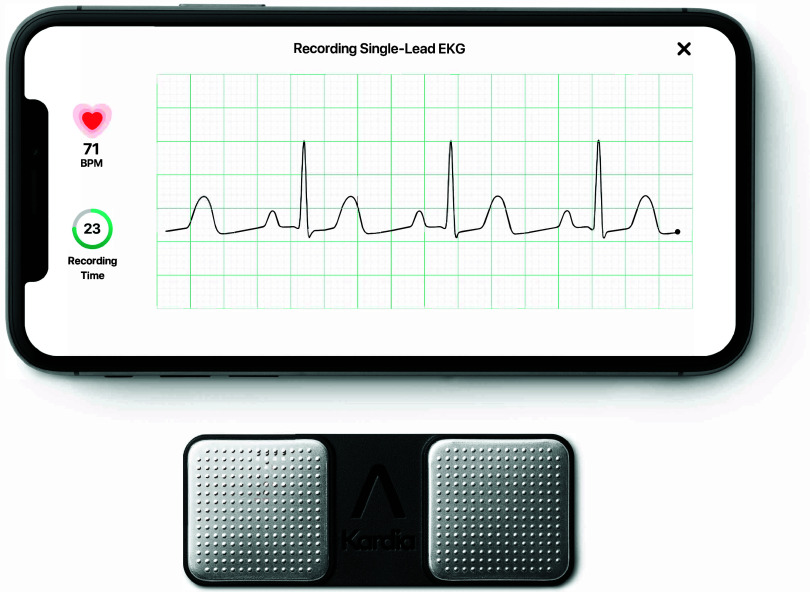
The KardiaMobile single-lead ECG device. Photograph by AliveCor, Mountain View, CA

The KardiaMobile (AliveCor, Mountain View, CA) is a handheld device used to record, store, and transfer 1L-ECG recordings. After activating the smartphone app, a 30-s 1L-ECG recording is obtained by holding the two electrodes with the fingers of both hands (see Figure 1). The connected smartphone app provides direct visual feedback. A built-in AF detection algorithm interprets the 1L-ECG recording for the presence of possible AF (see further below). The 1L-ECG recording can be shared as a PDF file using the smartphone app.

### Instructions for use

The GP cooperative instructed GPs during the general assembly of members and with an instruction leaflet to use the KardiaMobile when either an irregular pulse or a slow or fast pulse rate was noticed. This could be in both symptomatic and asymptomatic patients. The GP cooperative instructed GPs to consult the expert panel in the digital consultation platform whenever the result of the KardiaMobile’s built-in AF detection algorithm was abnormal (‘possible AF’; ‘unclassified’ or ‘unreadable’) or whenever GPs had doubts about the interpretation (also with a ‘normal’ algorithm result). Whenever GPs were certain of a 1L-ECG’s rhythm to be normal, and this was supported by the built-in algorithm, there was no further need to consult the expert panel.

### Outcomes

#### Digital consultation platform

In describing the use of the digital consultation platform, we reported the proportion of cases in which the expert panel could make a definitive diagnosis based on the shared 1L-ECG recording(s) as the primary outcome. We reported the time in which the GPs received an answer from the cardiologist, as assessed from the time stamps of the shared 1L-ECG and the first message from the expert panel in response to that case, as a secondary outcome.

#### Online questionnaire

1. GPs’ user experiences, regarding:
  - The KardiaMobile: its added value and its ease of use for rhythm diagnostics in daily primary care practice
  - The consultation platform: the added value of participating in the consultation platform and the usefulness of the expert panel’s responses and whether they replied in a timely manner
2. The perceived effects of this 1L-ECG implementation project on provided health care
3. GPs’ perspectives for future use of the KardiaMobile and consultation platform

### Data collection

#### Digital consultation platform

Two investigators (EK and JH) collected all 1L-ECG recordings and corresponding cases as shared by GPs in the digital consultation platform between December 2019 and January 2021. We extracted the following data from the cases shared in the digital consultation platform: the reasons for patients consulting their GP, the expert panel’s response time, the primary diagnosis according to the expert panel, and – whenever available – the recommendations provided by the expert panel regarding (a) additional diagnostics and (b) therapeutic interventions. Due to the observational nature of this study, the expert panel did not (consistently) report being able to make a definite diagnosis on the provided 1L-ECG. Therefore, this sometimes had to be derived from the context (eg, whenever the expert panel asked for an additional 12L-ECG for confirmation). To minimize observer bias in the data extraction process and interpretation, two investigators (EK and MdK) extracted all data in parallel, resolving discrepancies through discussion. Because this study was performed at the discretion of the GP cooperative, who aimed at evaluating this project after one year, no sample size was calculated.

### Online survey

#### Formatting the survey

We developed an online questionnaire using input from members of the GP cooperative and the Department of General Practice at the Amsterdam UMC. A pilot was subsequently held among five independent GPs to obtain user feedback, from which we formatted a final survey (see Supplemental Methods for the survey questions).

#### Survey participants

In January 2021, we invited GPs who had entered the consultation platform in Siilo (*n* = 98) for an online questionnaire by email. A generic link gave GPs access to an anonymous online secured survey (LimeSurvey, Amsterdam, the Netherlands). We reminded GPs via email, in the consultation platform, and during the general meeting of the GP cooperative. We closed the online survey after six weeks. We approached GPs who had not participated in the group chat in Siilo (*n* = 105) by email to investigate their reasons for not participating.

### Privacy

GPs submitted the 1L-ECG recordings in the digital consultation platform with de-identified patient information. Names of GPs and members of the expert panel were further de-identified during data collection. GPs and the expert panel were notified of this procedure and were given an OPT-OUT option. The online questionnaire was anonymous by design.

### Statistical analysis

Discrete and nominal variables are described as frequencies and percentages and continuous variables as mean ± standard deviation (SD). Non-normally distributed numerical data are noted as median with interquartile range (IQR). All statistical analyses were performed using SPSS version 26 (IBM Corp. Released 2019. IBM SPSS Statistics for Windows, Version 26.0. Armonk, NY: IBM Corp.).

## Results

### Digital consultation platform

**Figure 2.**
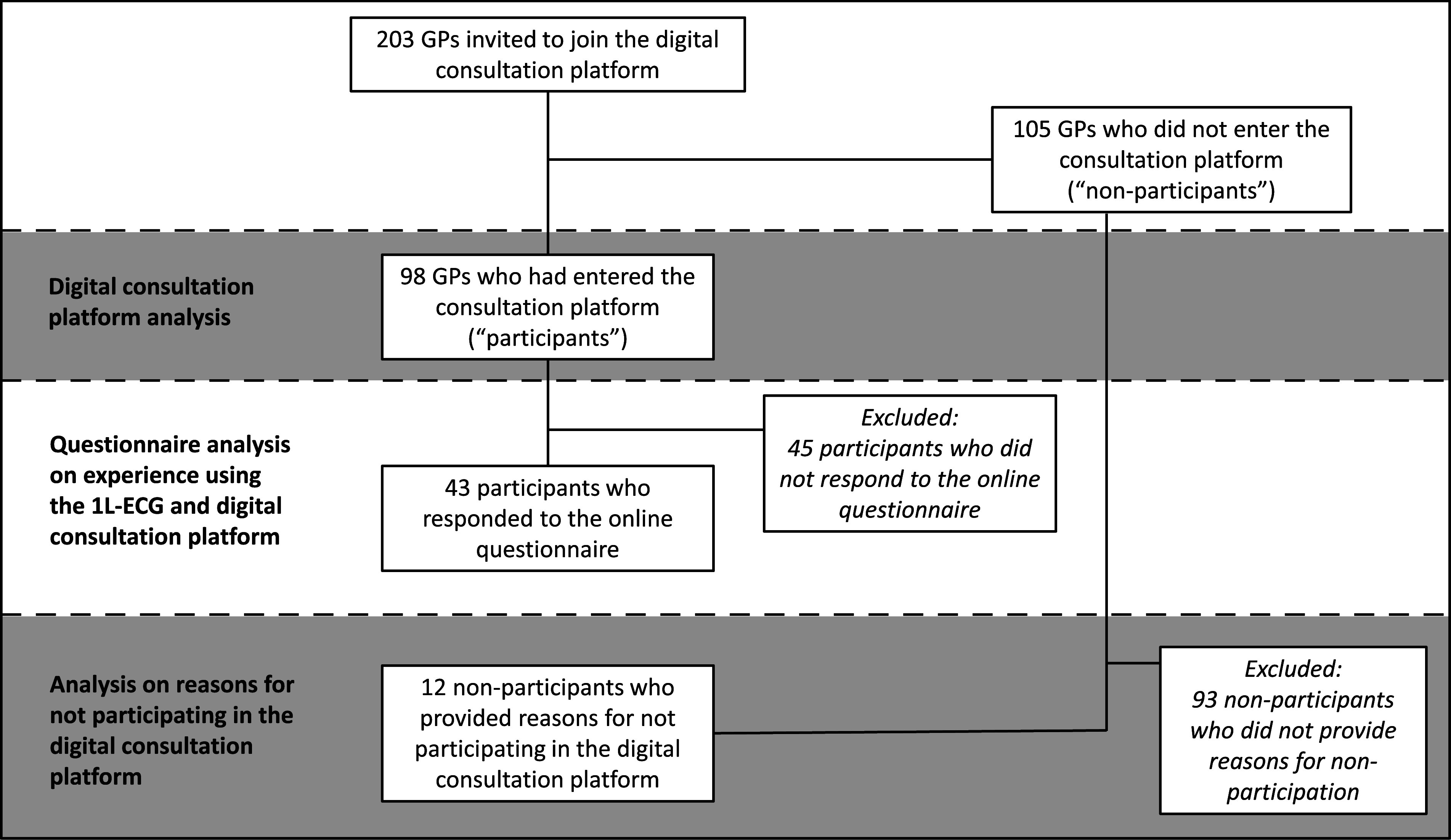
Flowchart showing the inclusion of GPs

In January 2021, 98 GPs (out of 203 invited) had participated in the digital consultation platform (see Flowchart in Figure 2). Of these 98 GPs, 48 (49%) had shared at least one case with the expert panel. From December 13, 2019, up until January 31, 2021, these 48 GPs had shared a total of 161 1L-ECGs in 156 patients in the digital consultation platform. Patients mostly experienced palpitations (37.4%), and dyspnea (on exertion) (21.6%) were asymptomatic (20.1%) or experienced chest pain (10.8%), dizziness (10.8%), and/or fatigue (7.9%).

The expert panel was able to provide a definitive ECG interpretation in 130 of the 156 patients (83.3%; 128 based on the first shared 1L-ECG, 2 additional cases based on the second shared 1L-ECG) (see Table 1). The most frequent rhythm diagnoses were sinus rhythm (*n* = 68; 43.6%), followed by AF or flutter (*n* = 38; 24.4%). The expert panel’s response time for the first 1L-ECG shared of the 130 patients with a definitive rhythm diagnosis based on 1L-ECG was a median of 4 min (IQR: 2–18).

**Table 1.** Interpretation of shared cases

Interpretation (*n* = 156)	*n* (%)
Certain interpretation	130 (83.3)
- Sinus rhythm	68 (43.6)
- Atrial fibrillation or flutter	38 (24.4)
- Sinus tachycardia	18 (11.5)
- Supraventricular tachycardia	5 (3.2)
- Other	1 (0.6)
Uncertain interpretation	26 (16.7)
Additional findings among interpretable cases (*n* = 130)	
Extrasystoles	47 (36.2)
- PVC	23 (17.7)
- PAC	23 (17.7)
- Both PACs and PVCs	1 (0.8)
Intraventricular conduction delay	10 (7.7)

1L-ECG = single-lead electrocardiogram; PVC = premature ventricular complex; PAC = premature atrial complex.

The expert panel provided a diagnostic recommendation in 87 cases: 55.8% (see Supplemental Table 1). Mostly, they recommended a 12L-ECG (*n* = 30; 34.5%). The expert panel provided treatment recommendations in 49 cases (31.4%), mostly related to pharmacologic rate control (*n* = 25; 51.0%).

### Online questionnaire

In total, 43 out of 98 (44%) participating GPs completed the questionnaire. Thirty-one (72%) were female, and 17 (39.5%) had access to a 12L-ECG device at their own practice. Responders had on average 16.6 (SD ± 6.9) years of work experience and worked 3.4 (SD ± 0.6) days a week. Twenty-four (56%) recorded fewer than 1 1L-ECG recordings per month, 18 (42%) in 1–5 per month, and 1 (2%) in 6–10 per month.

Most responding GPs (*n* = 39; 91%) actively used the consultation platform, either by sharing cases themselves (*n* = 27; 63%) or because they were interested in the cases shared by colleagues (see Supplemental Table 2). GPs indicated that they had shared cases in the consultation platform mostly because the built-in AF detection algorithm’s result was abnormal (*n* = 21; 78%) (see Supplemental Table 3). Remarkably, most of these 27 GPs also indicated that they had not shared all 1L-ECG recordings with an abnormal algorithm result, as opposed to the advice given by the ROHA *(*see Supplemental Table 4). This contrasted with the fact that most of the 43 participating GPs indicated not to feel any thresholds for sharing casuistry in the consultation platform (*n* = 32; 74%) (see Supplemental Table 5).

#### GPs’ user experiences

**Figure 3.**
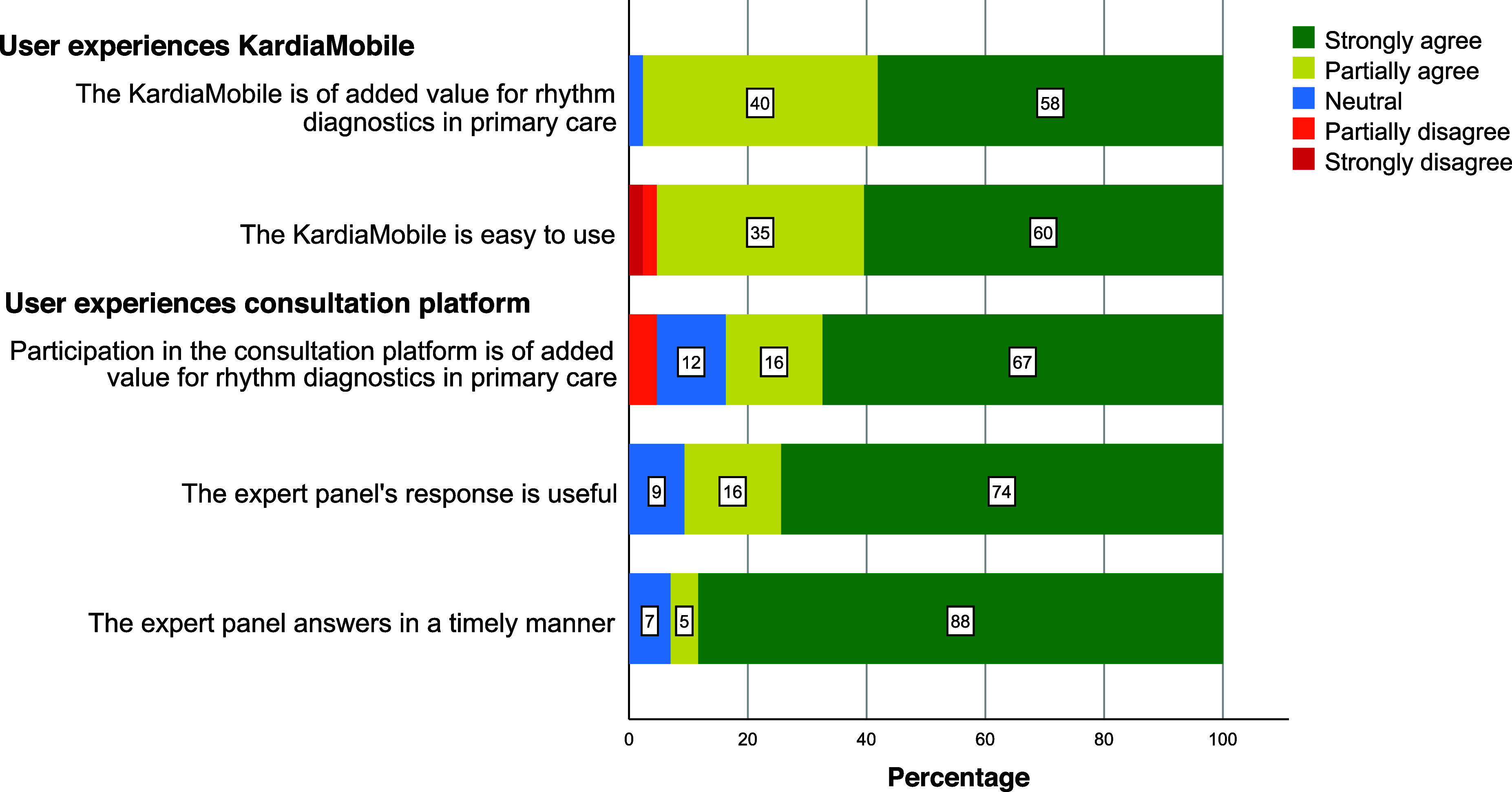
GP experiences using the KardiaMobile and the digital consultation panel (*n* = 43)

Figure 3 shows that responding GPs regarded the KardiaMobile as both of added value for rhythm diagnostics in primary care (*n* = 42; 98%) and easy to use (*n* = 41; 95%).

Thirty-six GPs (84%) indicated to think that the consultation platform was of added value for rhythm diagnostics in primary care and 39 (91%) thought the expert panel’s response was useful. Most GPs also appreciated the expert panel’s timely response (*n* = 40; 93%).

#### Perceived effects of this 1L-ECG implementation project on provided health care

The majority of the respondents indicated to think that this 1L-ECG implementation project improved the quality of care for patients with cardiac arrhythmias and improved diagnostic efficiency (see Supplemental Figure 1). Most responding GPs (*n* = 32; 74%) felt the KardiaMobile helped to avoid using other cardiac diagnostics, like 12L-ECGs (not in figure). Most GPs (*n* = 35; 81%) also (strongly) agreed with the statement that participating in this project improved their ability to diagnose or rule out AF on an ECG recording (both 1-lead and 12-lead). This 1L-ECG implementation project did not evidently affect the perceived workload for GPs. Seventeen (40%) respondents expected a reduced number of referrals to a cardiologist.

#### GPs’ perspectives on future use

Most (*n* = 42; 98%) responding GPs had a positive attitude toward the use of 1L-ECGs in primary care (see Supplemental Table 6). Twenty-four (56%) of these GPs intended to keep using the KardiaMobile in the future, regardless of the availability of a digital consultation platform. Factors that would help in adopting 1L-ECG devices were appropriate financial compensation, private sharing options with the expert panel, adoption of 1L-ECGs in national guidelines, and solving technical issues. Technical problems, for example, the troublesome connection of the 1L-ECG device to the smartphone app and poor signal quality, were demotivating and time-consuming.

#### GPs who did not participate in the project

From the 105 GPs who did not participate in the consultation platform’s group chat, we obtained a reason for not participating from 12 GPs. Four GPs reported never to have used the KardiaMobile because ‘it disappeared in a bottom drawer’. One said the KardiaMobile device malfunctioned, one was not able to install and navigate the KardiaMobile’s smartphone app, and one GP, not having used both the KardiaMobile and Siilo app, felt GPs were already flooded with tasks, but he also did not know of the existence of the Siilo app.

From other GPs who did use the KardiaMobile but never entered the Siilo app, two reported they did not know of the existence of a group chat, one said not to have been invited, one reported technical issues with Siilo, and one had no questions regarding the 1L-ECG recordings or referred to the specialist directly.

## Discussion

### Principal findings

In a GP cooperative-initiated real-life implementation project of a handheld 1L-ECG device (KardiaMobile), supported by a digital consultation platform, the expert panel reported being able to make a diagnosis in 83.3% of shared 1L-ECG recordings within a median of 4 min. A group of early-adopting GPs considered both the 1L-ECG device and the digital consultation platform as useful tools in daily clinical practice for rhythm diagnostics. GPs experienced the combined introduction of the 1L-ECG device and digital consultation platform to have a positive impact on the quality of care for patients with (suspected) cardiac arrhythmias and diagnostic efficiency, without evidently increasing the workload for GPs.

### Strengths and weaknesses of the study

In this study, we had unrestricted access to the 1L-ECG recordings and platform use of a group of early-adopting GPs within a larger GP cooperative. The observational nature of us documenting a real-life, GP cooperative-initiated implementation of a digital innovation, likely increased the relevance of our findings to GPs or GP cooperatives who wish to implement such a project in similar settings.

Several limitations of this study need to be addressed. First, we investigated a selected group of GPs who entered the consultation platform, potentially limiting the generalizability of results. While we were able to present key characteristics of participating GPs in our questionnaire, we were unable to compare these to the broader Amsterdam GP community. Unfortunately, it was not possible to gather more information on why GPs did not enter the consultation platform. Nevertheless, it appeared that there was a continuous increase in coverage across the GP cooperative in the years after this study, ranging from 62 participants in the consultation platform in April 2020 to 145 in May 2022. The COVID-19 pandemic may (partly) explain the initial relatively slow growth of participating GPs within this project.

Second, for data collection, we were dependent on the cases and information that GPs shared in the consultation platform. It is unknown how many and what kind of cases were not shared. Third, as is common in questionnaires, a selection bias may be present. GPs interested in cardiovascular diseases, enthusiastic about the KardiaMobile or otherwise motivated, were more likely to respond. Fourth, it is uncertain from our data to what extent our results are influenced by the use of KardiaMobile alone rather than other 1L-ECG devices.

Fifth, because of the observational nature of our research, we have no information on the exact reason(s) why the expert panel was unable to interpret certain 1L-ECGs, only that they requested a new one, asked for further cardiological work-up, or expressed their doubts in any other way.

Last, since ‘proportion of interpretable ECG cases’ and ‘response time’ are not commonly used outcome measures, how plausible they may seem, their validity and thereby clinical relevance are unknown. We also did not test the reliability of the expert panel’s interpretation with an external reference standard.

### Findings in the context of current literature

Selder and colleagues analyzed the use of the KardiaMobile in the ‘Hartwacht Arrhythmias’ program (Selder *et al*., 2019). In this program, outpatient cardiology patients received a KardiaMobile at the discretion of their cardiologists. From all 2434 1L-ECG recordings labeled as ‘possible AF’, ‘unclassified’, or ‘unreadable’ by the built-in AF detection algorithm, cardiologists reported being able to interpret 2076 (85.3%) recordings. This result is consistent with our results.

Little is known about digital consultation platforms in current medical literature. One recent observational study, also from the Netherlands, evaluated a low-threshold interdisciplinary consultation platform (Sanavro *et al*., 2022). The emergence of these consultation platforms initiated by physicians, as well as the rapid growth of use, can be seen as indicative of an unfilled need of healthcare professionals for a low-threshold consultation option. Interestingly, the mean response time was 76 min in this study, while ours had a median of 4 min. This may be explained because of the small-scale design of our app, with GPs and experts all working within the same region, often knowing each other personally. Furthermore, in the digital consultation platform we present here, experts were only asked to assess 1L-ECGs, while in the study by Sanavro *et al*., questions had a wide variety, as was the degree of difficulty of these questions.

To our knowledge, this is the first study to evaluate real-world experiences of early-adopting GPs with the use of the KardiaMobile in combination with a digital consultation platform in daily clinical practice. In 2019, Godin *et al.* studied the feasibility of the KardiaMobile as a diagnostic tool for AF screening in Canadian primary care (Godin *et al*., 2019). Using a questionnaire, they evaluated GPs’ experiences using the KardiaMobile and found a high perceived clinical value (94%) and high perception of the general ease to integrate the KardiaMobile into routine practice (89%). Although these GPs did not have access to a consultation platform, these results largely correspond with our findings.

Another study evaluating GPs’ perspectives on the use of a digital consultation platform for teledermatology also found positive results (Tensen *et al*., 2023). Most GPs would use the platform again and/or would recommend the platform to a colleague. Tensen *et al*. also found technical issues to be an important barrier experienced by GPs (human-computer interface and interoperability issues on the telemedicine platform).

We also found that an important part of participating GPs (*n* = 17; 40%) expected a reduced number of referrals to cardiologists because of this project. This is in line with results from Wilson *et al*. who showed that a low-threshold specialist consultation method using a telephone service prevented 60% of referrals (Wilson *et al*., 2016).

Last, respondents to our questionnaire indicated not to have experienced an evident impact on their workload. It is unknown what caused this perception; however, it might be this is caused by the lack of structured consultation options in usual care whereby telephonic consultation could take more time (see Organization of (primary) health care in the Netherlands).

### Clinical relevance

Diagnosing cardiac arrhythmias, in particular AF, can be difficult in daily primary care practice. Not all GPs possess a 12L-ECG device, and whenever they do, producing a 12L-ECG recording during symptoms can be difficult due to logistical barriers such as during house visits. Handheld 1L-ECG devices are promising tools to overcome these limitations due to their pocket-sized format, usability, and high diagnostic accuracy when interpreted by expert readers. Because previous research showed that GPs cannot reliably diagnose AF on a 1L-ECG recording, it is important for GPs to have the opportunity to consult an ECG expert (Karregat *et al*., 2021).

In this observational study, we showed that an expert panel reported being able to interpret more than 8 out of 10 1L-ECG recordings shared by GPs in a digital consultation platform. Given that there is no commonly accepted cut-off value for this outcome, the clinical relevance of this consultation platform was difficult to be assessed by this outcome alone. However, given the results of the questionnaire with generally positive feedback from participating GPs, these combined results are an indication that such a platform could result in clinical benefit. Because GPs were instructed only to share 1L-ECG recordings whenever they had doubts about the interpretation, as well as those that were otherwise classified as abnormal by the system’s algorithms, the net clinical benefit of the KardiaMobile is likely higher.

We also showed that early-adopting GPs who used the KardiaMobile 1L-ECG device in a real-life setting were enthusiastic of its use and potential value for daily clinical practice. Furthermore, we found these GPs perceived a digital consultation platform to be easy and useful for instantly obtaining 1L-ECG interpretation support. Despite the consultation platform’s excellent reviews, some GPs stated to keep using the KardiaMobile even without the availability of a consultation platform. Moreover, GPs felt participating in this project had a positive impact on their ability to diagnose or rule out AF on ECGs. The positive effects on health care of this learning effect may well increase with further adoption by more GPs and with prolonged participation in the consultation app.

The global use of 1L-ECG devices is growing. This is manifested by an increasing number of manufacturers introducing 1L-ECG products (eg, Apple, Fitbit, Samsung, etc.). Another indication is that 1L-ECGs are included in recent guidelines, such as the 2020 European Society of Cardiology guidelines for diagnosis and management of AF, in which a physician reviewed 1L-ECG recording is accepted as a valid diagnostic tool for AF. These developments highlight the importance of investigating the user experiences of important stakeholders.

This research shows a simple implementation project is received enthusiastically by early-adopting GPs, who perceived gains in multiple healthcare domains for all stakeholders: both patients and GPs, but also the healthcare system itself.

Although we lack a comprehensive view on reasons why GPs would not use the KardiaMobile and/or the consultation platform, our responders did mention a number of aspects important for the successful implementation of a 1L-ECG implementation project like this, such as support in solving technical difficulties and adoption of 1L-ECGs in national guidelines. It is important to address these issues and support GPs in addressing them.

For other GP cooperatives considering the implementation of a similar project, it is important to consider that GPs (and the expert panel) in the Amsterdam region are all in the possession of a mobile phone, email address, and good Internet connection. In other (low-resource) settings, these circumstances might be different, hindering a smooth implementation.

### Unanswered questions and future research

To stimulate the adoption of 1L-ECG devices and a supportive expert consultation platform, it will be important to gain more insights into reasons of GPs who are not motivated using these tools. Furthermore, it is important to investigate the perspectives of both GPs and cardiologists regarding the position of 1L-ECG devices in relation to 12-lead ECG machines. Patients’ perspectives regarding 1L-ECG devices are another subject of future study. More research is also needed to assess the effects on the incidence of AF (and other cardiac arrhythmias), the impact on health care, and the cost-effectiveness of this project. Finally, 1L-ECG recordings have been validated for diagnosing AF. However, in clinical practice – such as in our study – they have the potential to detect other (potentially relevant) ECG abnormalities. Future research should investigate the accuracy and the clinical impact of these findings.

## Conclusion

An expert panel felt being able to interpret most KardiaMobile 1L-ECG recordings as shared by GPs in a digital consultation platform. GPs participating in an online consultation platform to share potentially abnormal 1L-ECG recordings considered the KardiaMobile 1L-ECG device a useful tool. These GPs also considered a digital consultation platform to be of added value for rhythm diagnostics in daily primary care practice. These findings are relevant for GPs and cardiologists exploring new, low-threshold diagnostic pathways for diagnosis of AF or looking into an improved collaboration between primary and secondary care for patients with a suspected cardiac arrhythmia.

## Supporting information

Karregat et al. supplementary material Karregat et al. supplementary material

## Data Availability

Our data are available upon reasonable written request.

## Supplementary material

The supplementary material for this article can be found at https://doi.org/10.1017/S1463423624000057.

## Patient and public involvement

Patients and the public were not involved in the design or conduct of this study.
