# Supplementary material for: Evaluation of the introduction of a single-lead ECG device and digital cardiologist consultation platform among general practitioners in the Netherlands
Source: Prim Health Care Res Dev. 2024 Apr 18;25:e18. doi: 10.1017/S1463423624000057 (PMC11091535; doi:10.1017/S1463423624000057)
Supplement: Karregat et al. supplementary material [file S1463423624000057sup001.docx]

**Supplemental material**

**Supplemental Methods:**

STROBE Statement—checklist of items that should be included in reports of observational studies

|  | Item No. | Recommendation | Page  No. | Relevant text from manuscript |
| --- | --- | --- | --- | --- |
| **Title and abstract** | 1 | (*a*) Indicate the study’s design with a commonly used term in the title or the abstract | 1-2 | “Methods” section of abstract |
|  |  | (*b*) Provide in the abstract an informative and balanced summary of what was done and what was found | 1-.2 | “Methods” and “Findings” section of abstract |
| Introduction | | | |  |
| Background/rationale | 2 | Explain the scientific background and rationale for the investigation being reported | 3-4 | “Introduction” |
| Objectives | 3 | State specific objectives, including any prespecified hypotheses | 4 | Last paragraph of introduction: aims |
| Methods | | | |  |
| Study design | 4 | Present key elements of study design early in the paper | 5 | “study design” paragraph |
| Setting | 5 | Describe the setting, locations, and relevant dates, including periods of recruitment, exposure, follow-up, and data collection | 5-11 | “Organiation of (primary) health care in the Netherlands”, “1L-ECG implementation project”; “Expert panel”, “the KardiaMobile device and smartphone app”, “instruction for use” and “data collection” paragraphs |
| Participants | 6 | *Cross-sectional study*—Give the eligibility criteria, and the sources and methods of selection of participants | 6-7; 10-11 | Observational part: “1L-ECG implementation project and participating GPs” and for the survey: “data collection” |
| Variables | 7 | Clearly define all outcomes, exposures, predictors, potential confounders, and effect modifiers. Give diagnostic criteria, if applicable | 8-9 | “Outcomes” paragraph |
| Data sources/ measurement | 8* | For each variable of interest, give sources of data and details of methods of assessment (measurement). Describe comparability of assessment methods if there is more than one group | 9-10 | “digital consultation platform” and “formatting the survey” sections in paragraph “data collection” |
| Bias | 9 | Describe any efforts to address potential sources of bias | 9-10 | “data collection” section |
| Study size | 10 | Explain how the study size was arrived at | 9-10 | “digital consultation platform” section under ‘data collection’ |

Continued on next page

| Quantitative variables | 11 | Explain how quantitative variables were handled in the analyses. If applicable, describe which groupings were chosen and why | 11 | “statistical analysis” paragraph |
| --- | --- | --- | --- | --- |
| Statistical methods | 12 | (*a*) Describe all statistical methods, including those used to control for confounding | 11 | “statistical analysis” paragraph |
|  |  | (*b*) Describe any methods used to examine subgroups and interactions | N.A. |  |
|  |  | (*c*) Explain how missing data were addressed | N.A. |  |
|  |  | *Cross-sectional study*—If applicable, describe analytical methods taking account of sampling strategy | N.A. |  |
|  |  | (*e*) Describe any sensitivity analyses | N.A. |  |
| Results | | | | |
| Participants | 13* | (a) Report numbers of individuals at each stage of study—eg numbers potentially eligible, examined for eligibility, confirmed eligible, included in the study, completing follow-up, and analysed | 12-13 | First paragraphs from “Digital consultation platform” and “online questionnaire” sections in Results (and Flowchart figure 2). |
|  |  | (b) Give reasons for non-participation at each stage | 15 | “GPs who did not participate in the project” |
|  |  | (c) Consider use of a flow diagram | Fig.2 | Figure 2 |
| Descriptive data | 14* | (a) Give characteristics of study participants (eg demographic, clinical, social) and information on exposures and potential confounders | 13 | First paragraph from “online questionnaire” section in Results. |
|  |  | (b) Indicate number of participants with missing data for each variable of interest | N.A. |  |
| Outcome data | 15* | *Cross-sectional study—*Report numbers of outcome events or summary measures | 12 | “Digital consultation platform”-section in Results. |
| Main results | 16 | (*a*) Give unadjusted estimates and, if applicable, confounder-adjusted estimates and their precision (eg, 95% confidence interval). Make clear which confounders were adjusted for and why they were included | N.A. |  |
|  |  | (*b*) Report category boundaries when continuous variables were categorized | 12 | “(IQR: 2-18)” |
|  |  | (*c*) If relevant, consider translating estimates of relative risk into absolute risk for a meaningful time period | N.A. |  |

| Other analyses | 17 | Report other analyses done—eg analyses of subgroups and interactions, and sensitivity analyses | N.A. |  |
| --- | --- | --- | --- | --- |
| Discussion | | | | |
| Key results | 18 | Summarise key results with reference to study objectives | 16 | “principal findings” section in discussion |
| Limitations | 19 | Discuss limitations of the study, taking into account sources of potential bias or imprecision. Discuss both direction and magnitude of any potential bias | 16-17 | “strengths and weaknesses of the study” |
| Interpretation | 20 | Give a cautious overall interpretation of results considering objectives, limitations, multiplicity of analyses, results from similar studies, and other relevant evidence | 18-19 | “Findings in the context of current literature” |
| Generalisability | 21 | Discuss the generalisability (external validity) of the study results | 19-21 | “clinical relevance” |
| Other information | |  | | |
| Funding | 22 | Give the source of funding and the role of the funders for the present study and, if applicable, for the original study on which the present article is based | 23 | “Financial support” section under “Declarations”. |

*Give information separately for cases and controls in case-control studies and, if applicable, for exposed and unexposed groups in cohort and cross-sectional studies.

**Note:** An Explanation and Elaboration article discusses each checklist item and gives methodological background and published examples of transparent reporting. The STROBE checklist is best used in conjunction with this article (freely available on the Web sites of PLoS Medicine at http://www.plosmedicine.org/, Annals of Internal Medicine at http://www.annals.org/, and Epidemiology at http://www.epidem.com/). Information on the STROBE Initiative is available at www.strobe-statement.org.

**Supplemental Methods:**

**English translation of the questionnaire provided to general practitioners who participated in the online consultation platform**

| **Question** | **Answer options**  **(when applicable)** | **Answer type** |
| --- | --- | --- |
| *The following questions are meant to assess the characteristics of GPs who responded to our questionnaire* | | |
| Are you male or female? | - Male - Female | RB |
| For how many years have you been working as a GP? |  | NR |
| In what part of the city is your practice located? | - City Center - New West - North | RB |
| Do you have a 12-lead ECG device at your practice? | - Yes - No | RB |
| What percentage of your patients are aged 65 years or older? (estimation)? |  | NR |
| How many days per week do you work at your practice? | - 1 - 2 - 3 - 4 - 5 | RB |
| *The following questions are on your experience with using the KardiaMobile single-lead ECG for primary care patients* | | |
| How many KardiaMobile recordings did you make since the start of the project? | - None - Fewer than 1 per month - 1-5 per month - 6-10 per month - More than 10 per month | RB |
| Which symptoms as reported by a patient prompted you to use your KardiaMobile? | - Palpitations - Fatigue - Dizziness - Dyspnea - Chest pain - No particular symptom, but finding an irregular pulse during physical examination | CB |
| For what other indications did you use the KardiaMobile? |  | FT |
| Do other colleagues in your practice also use your KardiaMobile? | - No - Yes, practice nurse - Yes, practice assistant - Yes, GP trainee - Yes, other GPs - Other | CB |
| If you selected ‘other’, who used the KardiaMobile? |  | FT |
| I think using the KardiaMobile is easy | - Strongly disagree - Partially disagree - Neutral - Partially agree - Strongly agree | RB |
| Could you elaborate on your previous answer? |  | FT |
| I think the KardiaMobile is of additional value for rhythm diagnosis in primary care | - Strongly disagree - Partially disagree - Neutral - Partially agree - Strongly agree | RB |
| Could you elaborate on your previous answer? |  | FT |
| Were you a participant in the Siilo digital consultation platform at any point? | - Yes - No | RB |
| If you selected ‘no’, why not? |  | FT |
| *The following questions are on the use of the Siilo digital consultation platform, and on sharing ECG recordings within the group chat* | | |
| For how many months have you participated in de Siilo platform? |  | NR |
| What best describes your situation? | - Active contributor (shared one or more cases) - Active participant in (one or more) discussions on cases shared by other GPs - Observed shared cases/discussions with interest - No active interest in shared cases - Left the discussion platform | CB |
| If you indicated to have share one or more cases into the discussion platform, why did you do this? | - Abnormal algorithm reading - Non-abnormal algorithm reading, but I had doubts about the ECG interpretation - The case itself caused me to have questions (aside from the Kardia registration) for which I wished to consult a cardiologist - Other reason | CB |
| If ‘other reason’ was selected, what other reason? |  | FT |
| If you indicated to have left the discussion platform, for which reason did you leave? |  | FT |
| What percentage of cases with an abnormal algorithm reading do you estimate to have shared into the Siilo consultation platform? | - 0-20% - 21-40% - 41-60% - 61-80% - 81-100% | RB |
| Did you experience any thresholds/inhibitions for sharing an ECG reading into the discussion platform? | - No - Yes, too time consuming - Yes, enetering a case is too difficult - Yes, I am not sure what my question would be - Yes, I am embarrassed about a (perceived) lack of knowledge - Yes, other reason | CB |
| If ‘other reason’ was selected, what other reason? |  | FT |
| The expert team provided a timely response | - Strongly disagree - Partially disagree - Neutral - Partially agree - Strongly agree | RB |
| The expert team provided a good/helpful response | - Strongly disagree - Partially disagree - Neutral - Partially agree - Strongly agree | RB |
| I experienced participation in the Siilo digital consultation platform as having additional value for rhythm assessment in primary care | - Strongly disagree - Partially disagree - Neutral - Partially agree - Strongly agree | RB |
| If you wish, you are able to elaborate on your answer to the previous question |  | FT |
| *The following questions are on the influence of the project (reporting single-lead ECGs and participation in the Siilo digital consultation platform) on your ECG interpretation skills and your opinion on cardiac rhythm assessment in primary care* | | |
| Due to my participation in the project, I am better able to detect or rule out AF on ECG | - Strongly disagree - Partially disagree - Neutral - Partially agree - Strongly agree | RB |
| Through using the KardiaMobile device, have you been able to reduce the use of Holter or 12-lead ECG? | - Yes, based on the KardiaMobile alone - Yes, but only because the digital consultation platform was also available - Nee - Not sure | RB |
| If you wish, you are able to elaborate on your answer to the previous question |  | FT |
| *What effect do you think participation in the project has had on the following:* | | |
| Awareness of rhythm disorders | - Increased - Neutral - Decreased | RB |
| Quality of care for patients with a (suspected) rhythm disorder | - Increased - Neutral - Decreased | RB |
| Number of referrals to cardiologists | - Increased - Neutral - Decreased | RB |
| Workload for general practitioners | - Increased - Neutral - Decreased | RB |
| Application of the Dutch College of General Practitioners guidelines for atrial fibrillation | - Increased - Neutral - Decreased | RB |
| Patient centered care (as experienced by you as a general practitioner) | - Increased - Neutral - Decreased | RB |
| Patient satisfaction | - Increased - Neutral - Decreased | RB |
| Cost effectiveness | - Increased - Neutral - Decreased | RB |
| Timeliness of rhythm diagnosis | - Increased - Neutral - Decreased | RB |
| What do you think of (potentially) increasing the burden of care for atrial fibrillation towards general practitioners? | - Desirable - Neutral/no opinion - Undesirable | RB |
| If you wish, you are able to elaborate on your answer to the previous question |  | FT |
| The *following questions are on your vision on the future of diagnostic work-up for rhythm disorders in the context of using single-lead ECG in combination with a digital consultation platform for sharing rhythm recordings with a cardiologist* | | |
| I think I will keep using the KardiaMobile single-lead ECG for diagnosing rhythm disorders in primary care | - No - Yes, regardless of whether I can share recordings with colleagues (cardiologists or general practitioners) - Only if I can share recordings with colleagues (cardiologists or general practitioners) - Only if I can share recordings with a cardiologist in private - Other | RB |
| If you selected ‘Other’, please elaborate on the previous questions |  | FT |
| What is your vision on the future use of single-lead ECG |  | FT |
| Do you see any other preconditions for the continued use of single-lead ECG? | - Yes - No | RB |
| If you selected ‘Yes’, please elaborate on the preconditions for the continued use of single-lead ECG |  | FT |
| The KardiaMobile or similar single-lead ECGs could also be provided to a patient to be used (for prolonged periods of time) for paroxysmal symptoms such as palpitations, as an alternative for an event recorder. Would you use the KardiaMobile for this indication? | - No - Not sure - Yes, but only in patients for whom a regular event recorder is too burdensome - Yes, but only in patients with a long interval between symptoms - Yes, but only if a cardiological service provides monitoring for acute findings - Yes, in all cases described above | CB |
| If you wish, you are able to elaborate on your answer to the previous question |  | FT |
| *Thank you for participating in the survey* | | |

CB, checkbox (multiple options allowed); ECG, electrocardiogram; FT, free text; GP, general practitioner; NR, number; RB, radio button (one option allowed).

**Supplemental Data**

**Supplemental figure 1:** Perceived effects of this 1L-ECG implementation project according to responding GPs (n=43).


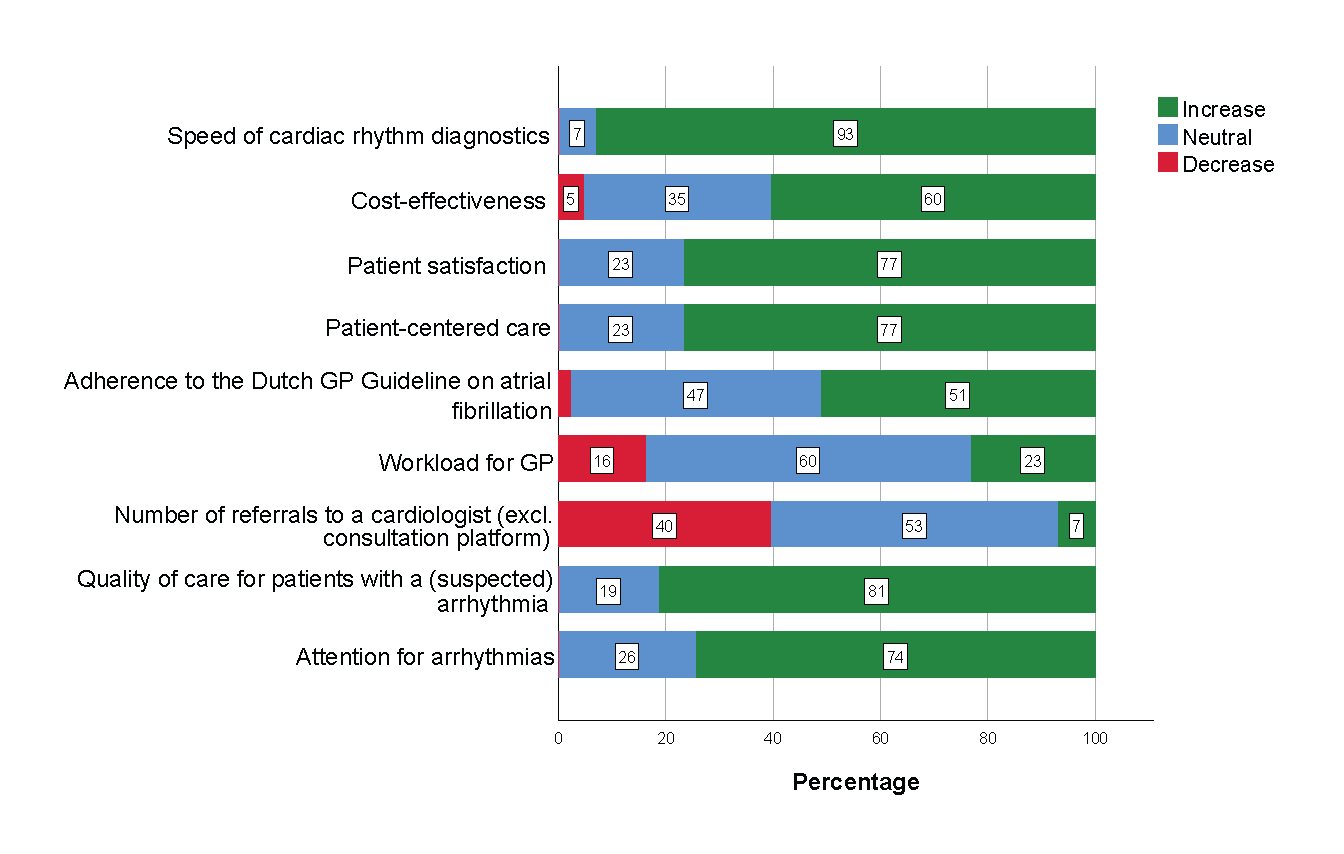


GP, general practitioner

**Supplemental table 1:** Number and proportion of additional diagnostic and/or treatment recommendations provided by the expert panel from a total of 156 shared cases.

|  | n (%) |
| --- | --- |
| Diagnostic recommendations | 87 (55.8) |
| - 12-lead ECG | 30 (34.5) |
| - Holter monitoring | 25 (28.7) |
| - Other^*^ | 24 (27.6) |
| - Referral to cardiologist for additional diagnostics | 18 (20.7) |
| - Echocardiography | 15 (17.2) |
| - Cycle ergometry | 7 (8.0) |
| - Event recording | 6 (6.9) |
| - No additional diagnostics required | 6 (6.9) |
| Treatment recommendations | 49 (31.4) |
| - Pharmacological rate control | 25 (51.0) |
| - Anticoagulant treatment | 16 (32.7) |
| - Referral to cardiologist for specialist treatment | 16 (32.7) |
| - No treatment required | 6 (12.2) |
| - Pharmacological rhythm control | 3 (6.1) |
| - Other | 2 (4.1) |

^*^“Other” diagnostics included laboratory research and not otherwise specified “heart failure investigations”.

The total number exceeds 100% because more than one recommendation was possible per case.

**Supplemental table 2:** How GPs label their participation in the consultation platform.

| **Consultation platform user description (n=43)** | **n (%)** |
| --- | --- |
| - Pro-active introducer of (one or more) cases | 27 (62.8%) |
| - Active participant of at least one discussion of shared cases by colleague GPs | 0 (0.0%) |
| - Interested follower of discussions and/or cases shared in the group chat | 27 (62.8%) |
| - Not actively interested in discussions or shared cases | 3 (7.0%) |
| - Left the group chat | 1 (2.3%) |
| More than one answer possible.  GP, general practitioner. |  |

**Supplemental table 3.** Reasons for consulting the expert panel in the consultation platform.

| **Reasons for sharing cases (n=27)** | **n (%)** |
| --- | --- |
| - Abnormal algorithm result | 21 (78%) |
| - Negative algorithm result, however doubt of interpretation | 6 (22%) |
| - The case raised questions (apart from the 1L-ECG recording) for which | 5 (19%) |
| - consultation of an expert was desirable |  |
| - Unknown | 1 (4%) |
| More than one answer possible |  |

**Supplemental table 4.** Self-reported estimated percentage of recordings with an abnormal algorithm reading shared in the online consultation platform (n=27)

| **Self-reported estimated percentage of recordings with an abnormal algorithm reading shared in the online consultation platform (n=27)** | **n (%)** |
| --- | --- |
| - 0-20% | 8 (30%) |
| - 21-40% | 4 (15%) |
| - 41-60% | 0 (0%) |
| - 61-80% | 3 (11%) |
| - 81-100% | 12 (44%) |

1L-ECG, single-lead electrocardiogram; GP, general practitioner.

**Supplemental table 5:** Thresholds experienced by GPs for sharing cases in the digital consultation platform.

| **Experienced thresholds for using the consultation platform? (n=43)** | **n (%)** |
| --- | --- |
| No | 32 (74%) |
| Yes* |  |
| Perceived lack of knowledge | 5 (12%) |
| Takes too much time | 4 (9%) |
| I find it hard to specify a question | 2 (5%) |
| Sharing a case in the consultation platform is too difficult | 0 (0%) |
| Other | 3 (7%) |
| *More than one answer possible  GP, general practitioner. |  |

**Supplemental table 6:** Future use of 1L-ECG devices by responding GPs.

| **I intend to keep using the KardiaMobile, or a comparable 1L-ECG device, for rhythm diagnostics (n=43)** | **n (%)** |
| --- | --- |
| Yes |  |
| Regardless of the availability of the consultation platform | 24 (56%) |
| Only whenever the consultation platform is available | 15 (35%) |
| Only whenever a privat-sharing option becomes available within the consultation  platform | 2 (5%) |
| No | 1 (2%) |
| Other: *"I would gladly keep using the device, but only whenever the device works better"*  *[GP mentioned having encountered troublesome connection to the mobile app]* | 1 (2%) |

GP, general practitioner.

**Abbreviations**

1L-ECG = single-lead electrocardiogram;

12L-ECG = 12-lead electrocardiogram;

AF = atrial fibrillation;

ECG = electrocardiogram;

GP = general practitioner;

IQR = interquartile range;

PAC = premature atrial complex;

PVC = premature ventricular complex;

ROHA = GP cooperative Amsterdam (in Dutch: Regionale Organisatie Huisartsen Amsterdam)

SD = standard deviation
